# IA和HAD方案诱导治疗成人初诊急性髓系白血病的疗效比较

**DOI:** 10.3760/cma.j.issn.0253-2727.2022.05.006

**Published:** 2022-05

**Authors:** 丛笑 张, 少伟 邱, 本法 宫, 晓媛 弓, 艳 李, 云涛 刘, 秋云 房, 广吉 张, 凯奇 刘, 春林 周, 述宁 魏, 冬 林, 兵城 刘, 迎 王, 营昌 秘, 辉 魏, 建祥 王

**Affiliations:** 中国医学科学院北京协和医学院血液病医院（中国医学科学院血液学研究所），实验血液学国家重点实验室，国家血液系统疾病临床医学研究中心，细胞生态海河实验室，天津 300020 State Key Laboratory of Experimental Hematology, National Clinical Research Center for Blood Diseases, Haihe Laboratory of Cell Ecosystem, Institute of Hematology & Blood Diseases Hospital, Chinese Academy of Medical Sciences & Peking Union Medical College, Tianjin 300020, China

**Keywords:** 白血病，髓样，急性, 高三尖杉酯碱, 伊达比星, 抗肿瘤联合化疗方案, 治疗结果, Leukemia, myeloid, acute, Homoharringtonine, Idarubicin, Antineoplastic combined chemotherapy protocols, Treatment outcome

## Abstract

**目的:**

比较IA和HAD方案诱导治疗成人初诊急性髓系白血病（AML）患者的疗效。

**方法:**

分析2014年5月至2019年11月在中国医学科学院血液病医院接受IA或HAD方案诱导化疗的199例成人初诊AML患者的完全缓解（CR）率、1个疗程微小残留病（MRD）转阴率、总生存（OS）和无复发生存（RFS）情况。评估年龄、初诊WBC、NPM1突变、FLT3-ITD突变、2017ELN危险度分层、第1次完全缓解（CR_1_）期造血干细胞移植（HSCT）和巩固化疗时使用大剂量阿糖胞苷（HDAC）对于不同诱导化疗组预后的影响。

**结果:**

199例患者中，男104例，女95例，中位年龄37（15～61）岁。90例患者接受IA诱导方案，109例接受HAD诱导方案。1个疗程诱导化疗后IA和HAD方案组的CR率分别为71.1％和63.3％（*P*＝0.245），流式细胞术MRD转阴率分别为53.3％和48.6％（*P*＝0.509）；3例患者诱导化疗1个疗程后60 d内死亡，IA组1例，HAD组2例；两组2年OS率分别为61.5％和62.1％（*P*＝0.835），2年RFS率分别为51.6％和57.8％（*P*＝0.291），差异均无统计学意义。多因素分析显示ELN危险度分层是两种诱导方案组的独立预后因素；CR_1_期HSCT是IA组患者OS和RFS的独立预后因素，对于HAD组患者，CR_1_期HSCT是RFS的独立预后因素，但不是OS的独立预后因素；年龄、高白细胞、NPM1突变和FLT3-ITD突变均无独立预后意义。

**结论:**

IA与HAD方案疗效相同，均是AML有效的治疗方案。

## Abstract (en)

**Objective:**

To compare the efficacy of two induction regimens, namely, idarubicin combined with cytarabine（IA）versus the combination of homoharringtonine, daunorubicin, and cytarabine（HAD）, in adult patients with newly diagnosed de novo acute myeloid leukemia（AML）.

**Methods:**

From May 2014 to November 2019, 199 patients diagnosed with AML receiving either the IA or HAD regimens were assessed for overall survival（OS）, relapse-free survival（RFS）, as well as the CR rate and the MRD negative rate after induction therapy. The differences in prognosis between the two induction therapy groups was assessed according to factors, including age, white blood cell（WBC）count, NPM1 mutation, FLT3-ITD mutation, 2017 ELN risk stratification, CR_1_ transplantation, and the use of high-dose cytarabine during consolidation therapy, etc.

**Results:**

Among the 199 patients, there were 104 males and 95 females, with a median age of 37（15–61）years. Ninety patients received the IA regimen, and 109 received the HAD regimen. Comparing the efficacy of the IA and HAD regimens, the CR rates after the first induction therapy were 71.1％ and 63.3％, respectively（*P*＝0.245）, and the MRD negative rates after the first induction therapy were 53.3％ and 48.6％, respectively（*P*＝0.509）. One patient in the IA group and two in the HAD group died within 60 days after induction. The two-year OS was 61.5％ and 70.6％, respectively（*P*＝0.835）, and the two-year RFS was 51.6％ and 57.8％, respectively（*P*＝0.291）. There were no statistically significant differences between the two groups. Multivariate analysis showed that the ELN risk stratification was an independent risk factor in both induction groups; CR_1_ HSCT was an independent prognostic factor for OS and RFS in the IA patients and for RFS in the HAD patients but not for OS in the HAD patients. Age, WBC level, NPM1 mutation, and FLT3-ITD mutation had no independent prognostic significance.

**Conclusion:**

The IA and HAD regimens were both effective induction regimens for AML patients.

蒽环类药物如伊达比星（IDA）、柔红霉素（DNR）联合阿糖胞苷（Ara-C）一直是治疗急性髓系白血病（AML）的标准疗法，60％～80％的成人AML患者可通过该诱导化疗方案获得完全缓解（CR）[1]。既往对IDA与DNR的疗效对比研究显示，与60～90 mg·m^−2^·d^−1^静脉给药3 d的DNR方案相比，标准剂量IA方案（12 mg）可以取得相当或者更好的诱导化疗效果[2]。高三尖杉酯碱（HHT）联合DA组成的HAD方案同样是AML的一线治疗方案[3]。本研究中，我们总结并分析了在我中心接受IDA联合标准剂量Ara-C诱导化疗方案治疗患者的预后情况，并与本中心之前接受HAD方案诱导化疗的患者队列进行对比分析。

## 病例与方法

1. 病例：在2014年5月至2019年11月就诊于中国医学科学院血液病医院白血病诊疗中心的1233例原发初治AML（非急性早幼粒细胞白血病）患者中，90例患者采用IA方案诱导化疗，109例患者采用HAD方案（标准剂量Ara-C）。患者均接受了骨髓细胞形态学、免疫表型分析、细胞遗传学、分子生物学、二代测序、流式细胞术微小残留病（MRD）检测。诊断符合文献[4]标准，所有病例根据2017欧洲白血病网（European LeukemiaNet，ELN）标准[5]进行危险度分层。

2. 治疗方案：IA组全部患者采用IDA与Ara-C联合方案进行诱导化疗，具体为：IDA 12 mg·m^−2^·d^−1^，静脉滴注，第1～3天；Ara-C 100 mg·m^−2^·d^−1^，静脉滴注，第1～7天。巩固化疗方案包括大剂量Ara-C（3 g·m^−2^·d^−1^，每12 h 1次静脉滴注，第1～3天）、DA（DNR 60 mg·m^−2^·d^−1^，静脉滴注，第1～3天；Ara-C 100 mg·m^−2^·d^−1^，静脉滴注，第1～7天）及HA（HHT 2.5 mg·m^−2^·d^−1^，静脉滴注，第1～7天；Ara-C 100 mg·m^−2^·d^−1^，静脉滴注，第1～7天）等。HAD组诱导方案为：HHT 2 mg·m^−2^·d^−1^，静脉滴注，第1～7天；DNR 40 mg·m^−2^·d^−1^，静脉滴注，第1～3天；Ara-C 100 mg·m^−2^·d^−1^，静脉滴注，第1～7天。巩固化疗方案包括大剂量Ara-C、DA、MA（米托蒽醌6 mg·m^−2^·d^−1^，静脉滴注，第1～3天；Ara-C 100 mg·m^−2^·d^−1^，静脉滴注，第1～7天）和中剂量Ara-C（1.5 g·m^−2^·d^−1^，每12 h 1次静脉滴注，第1～3天）。达到形态学CR后按照2017 ELN危险度分层选择不同的后续治疗方案，低危组与无合适供者的中、高危组患者进行巩固化疗，有合适供者的中、高危组患者选择接受造血干细胞移植（HSCT）治疗。

3. 疗效评价：疗效评估标准参考文献[6]。总生存（OS）时间指患者初次入院治疗之日起至死亡或末次随访的时间；无复发生存（RFS）时间指患者达到形态学CR之日起至原疾病复发、死亡或者末次随访的时间。

4. 随访：随访截止日期为2021年11月30日，随访资料来源于住院、门诊病历及电话随访记录。

5. 统计学处理：不同组别基线对比中性别比例对比使用二项分布检验，年龄对比使用独立样本*t*检验，血常规对比使用非参数检验，不同ELN危险度分布使用Pearson卡方检验。影响诱导化疗疗效的单因素分析使用Pearson卡方检验，多因素分析使用二元Logistic回归检验。用Kaplan-Meier法计算生存曲线。不同组别之间生存的比较使用Log-rank法检验。采用Cox回归模型进行多因素生存分析。采用SPSS 23.0.0与R 4.0.0进行统计分析。*P*<0.05为差异有统计学意义。

## 结果

1. 临床特征：199例原发初治AML患者中，男104例，女95例，中位年龄37（15～61）岁。入院中位WBC 19.67（0.74～296.34）×10^9^/L，HGB 84（38～138）g/L，PLT 43（5～317）×10^9^/L。NPM1突变率16.1％，FLT3-ITD突变率18.6％。55例患者在第1次完全缓解（CR_1_）期接受了HSCT。2017 ELN危险度分层：低危组97例（48.7％），中危组59例（29.6％），高危组43例（21.6％）。两组患者的临床特征见表1。中位随访时间33.6（2.1～76.4）个月。

**表1:** 199例原发初治急性髓系白血病患者临床基本特征

临床特征	IA组（90例）	HAD组（109例）	统计量	*P*值
年龄［岁，*M*（范围）］	39.5（15～61）	35（15～54）	−1.525	0.127
性别（例，男/女）	46/44	58/51	1.088	0.768
WBC［×10^9^/L，*M*（范围）］	20.27（0.76～252.71）	18.85（0.74～296.34）	−0.858	0.391
HGB［g/L，*M*（范围）］	86.5（38～138）	81（51～125）	−1.938	0.053
PLT［×10^9^/L，*M*（范围）］	40（5～259）	45（5～317）	−0.680	0.496
ELN危险度分组［例（％）］				0.344
低危组	47（52.2）	50（45.9）		
中危组	22（24.4）	37（33.9）	0.633	0.174
高危组	21（23.3）	22（20.2）	1.015	0.967
巩固方案含大剂量阿糖胞苷［例（％）］	10（11.1）	50（45.9）	0.131	<0.001
CR_1_期移植［例（％）］	32（35.6）	24（22.0）	1.954	0.035
随访时间［月，*M*（范围）］	27.8（2.1～33.7）	58.8（21.8～76.4）	−5.283	<0.001

注：IA：伊达比星+阿糖胞苷；HAD：高三尖杉酯碱+柔红霉素+阿糖胞苷；ELN：欧洲白血病网；CR_1_：第1次完全缓解

2. 诱导化疗疗效：全部199例患者中，诱导化疗1个疗程达CR 136例（68.3％），2个疗程达CR 33例（16.6％）。诱导1个疗程结束后60 d内3例患者死亡，IA组1例，HAD组2例。IA组和HAD组1个疗程诱导化疗后CR率分别为71.1％和63.3％（*OR*＝1.003, 95％*CI* 0.998～1.009，*P*＝0.245）；MRD转阴率分别为53.3％和48.6％（*OR*＝0.998，95％*CI* 0.993～1.004，*P*＝0.509），差异均无统计学意义。

对初诊高白细胞（WBC>50×10^9^/L）的患者进行分析，IA组和HAD组1个疗程CR率相对较低，但差异无统计学意义［48.1％（13/27）对60.0％（15/25），*OR*＝1.615，95％*CI* 0.538～4.853，*P*＝0.392］。

对于携带NPM1突变的患者，IA组和HAD组的1个疗程CR率分别为75.0％（9/12）和70.0％（14/20），差异无统计学意义（*OR*＝1.286，95％*CI* 0.255～6.492，*P*＝0.761）。携带FLT3-ITD突变的患者，无论是否合并NPM1突变，IA组和HAD组的1个疗程CR率均相对较低，但同样差异无统计学意义［45.4％（10/22）对40.0％（6/15），*OR*＝1.250，95％*CI* 0.330～4.731，*P*＝0.742］。1个疗程MRD转阴率趋势相同（NPM1组：*OR*＝0.611，95％*CI* 0.138～2.708，*P*＝0.780；FLT3-ITD组：*OR*＝0.361，95％*CI* 0.079～1.658，*P*＝0.329）。

3. 长期疗效：199例患者的2年OS率为61.2％，2年RFS率为54.6％。IA组2年OS率为61.5％（95％*CI* 51.3％～73.8％），2年RFS率为51.6％（95％*CI* 40.3％～66.2％）。HAD组2年OS率为62.1％（95％*CI* 53.6％～72.0％），2年RFS率为57.8％（95％*CI* 48.4％～68.9％），差异均无统计学意义（OS：*HR*＝0.951，95％*CI* 0.594～1.524，*P*＝0.835；RFS：*HR*＝0.778，95％*CI* 0.488～1.240，*P*＝0.291）（图1）。

**图1:**
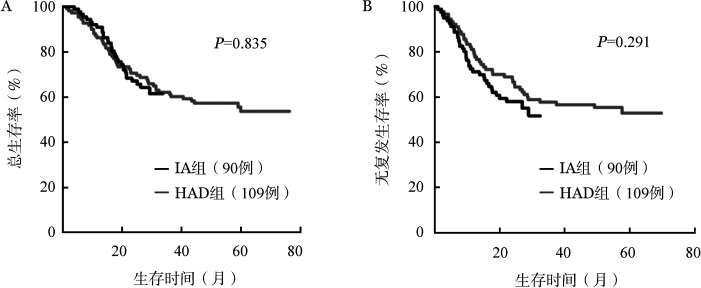
IA和HAD方案治疗急性髓系白血病患者的总生存（A）和无复发生存（B）曲线 IA：伊达比星+阿糖胞苷；HAD：高三尖杉酯碱+柔红霉素+阿糖胞苷

NPM1突变患者中，IA组和HAD组的2年OS率分别为83.3％和80.0％（*HR*＝1.185，95％*CI* 0.217～6.473，*P*＝0.844），2年RFS率分别为75.0％和83.3％（*HR*＝0.536，95％*CI* 0.133～2.153，*P*＝0.372）。FLT3-ITD突变患者中，IA组和HAD组的2年OS率分别为48.0％和33.3％（*HR*＝1.421，95％*CI* 0.598～3.378，*P*＝0.422），2年RFS率分别为51.3％和36.4％（*HR*＝1.014，95％*CI* 0.384～2.678，*P*＝0.977）。

4. 影响不同方案CR率和长生存的多因素分析：纳入年龄、初诊WBC、NPM1突变、FLT3-TID突变、ELN危险度分层、巩固方案是否含大剂量Ara-C、CR_1_期是否进行HSCT等因素对两方案组患者分别进行单因素分析。结果显示初诊WBC、FLT3-ITD突变、ELN危险度分层与患者1个疗程CR率和MRD转阴率相关；初诊WBC、FLT3-ITD突变、NPM1突变、ELN危险度分层、CR_1_期是否进行HSCT与患者OS和RFS相关（*P*值均<0.05）。将以上因素纳入Cox回归模型进行多因素分析。

通过多因素分析可以得出，ELN危险度分层是影响IA和HAD组患者首疗程化疗疗效的独立预后因素（*P*<0.001）（表2）；CR_1_期HSCT是IA组患者OS和RFS的独立预后因素（OS：*HR*＝0.372，95％*CI* 0.160～0.862，*P*＝0.021；RFS：*HR*＝0.216，95％*CI* 0.086～0.540，*P*＝0.001），对于HAD组患者，CR_1_期HSCT是RFS的独立预后因素（*HR*＝0.320，95％*CI* 0.124～0.828，*P*＝0.019），但不是OS的独立预后因素（*HR*＝0.441，95％*CI* 0.194～1.004，*P*＝0.051）（表3）。

**表2:** 影响急性髓系白血病患者首疗程化疗疗效的多因素分析（*P*值）

因素	IA组		HAD组	
	1个疗程CR	1个疗程MRD转阴	1个疗程CR	1个疗程MRD转阴
初诊WBC≥50×10^9^/L	0.650	0.363	0.298	0.132
FLT3-ITD突变	0.225	0.863	0.792	0.302
ELN危险度分层	<0.001	0.009	<0.001	0.001

注：IA：伊达比星+阿糖胞苷；HAD：高三尖杉酯碱+柔红霉素+阿糖胞苷；CR：完全缓解；MRD：微小残留病；ELN：欧洲白血病网

**表3:** 影响急性髓系白血病患者长期预后的多因素分析

因素	IA组				HAD组			
	OS		RFS		OS		RFS	
	*HR*(95％*CI*)	*P*值	*HR*(95％*CI*)	*P*值	*HR*(95％*CI*)	*P*值	*HR*(95％*CI*)	*P*值
初诊WBC≥50×10^9^/L	0.601（0.248～1.455）	0.206	0.835（0.367～1.896）	0.666	2.787，1.507～5.157	0.001	1.305，0.596～2.957	0.506
NPM1突变	0.361（0.080～1.626）	0.184	0.483（0.151～1.548）	0.221	0.551，0.180～1.683	0.296	0.543，0.180～1.640	0.279
FLT3-ITD突变	1.534（0.647～3.638）	0.331	1.401（0.571～3.439）	0.461	1.045，0.445～2.450	0.920	0.996，0.381～2.601	0.993
ELN危险度分组		0.012		0.032		0.002		0.005
CR_1_期移植	0.372（0.160～0.862）	0.021	0.216（0.086～0.540）	0.001	0.441，0.194～1.004	0.051	0.320，0.124～0.828	0.019

注：IA：伊达比星+阿糖胞苷；HAD：高三尖杉酯碱+柔红霉素+阿糖胞苷；OS：总生存；RFS：无复发生存；CR_1_：第1次完全缓解；ELN：欧洲白血病网

## 讨论

本研究中，我们观察了IA和HAD方案对原发初治AML患者的疗效。与国际标准的IA方案相比，我中心既往含标准剂量Ara-C的HAD诱导方案表现出相似的诱导缓解效率与生存率。

IDA是以DNR为基础改构而来的蒽环类药物，较DNR具有更好的亲脂性，从而可以延长其血浆半衰期[7]。并且相较于DNR，IDA具有更小的心脏毒性[8]。12 mg/m^2^×3 d是目前相关指南中IA诱导方案的建议剂量[3]，已有多个队列验证了12 mg IA诱导方案的有效性，Lee等[9]的队列包括15～65岁的AML患者，其1个疗程CR率为71.1％，4年OS率为51.1％，4年无事件生存（EFS）率为45.5％；Gardin等[10]所研究的50岁以上患者队列中，1个疗程CR率为69％，虽然没有转化为更长的OS时间（与DA组相比，*P*＝0.13），但可以获得更高的治愈率（16.6％比9.8％，*P*＝0.049）。

中国是首次使用HHT进行临床研究的国家[11]。HAD诱导化疗方案作为我国特色的AML治疗方案，被纳入我国AML指南[3],[12]。来自多中心的临床试验已证实，HAD方案与DA方案在治疗<60岁的年轻AML患者时，总体CR率和EFS率差异无统计学意义[13]。目前对比HAD方案与IA方案诱导疗效的临床研究较少。王涛等[14]的研究表明，对于携带FLT3-ITD单突变的AML患者，HAD三药联合方案比IA方案表现出更好的CR率与OS，但是对于合并NPM1突变的患者，这两种方案并未显示明显差异[14]。但是在我们的研究中，由于FLT3-ITD单突变患者样本量有限（18/37），无法对其进行分组统计。

目前认为初诊高白细胞（WBC>100×10^9^/L）和高龄患者（>60岁）均提示AML的不良预后[3]。但是在我们的队列中，无论在IA组还是HAD组，初诊高白细胞和非高白细胞患者的诱导治疗疗效、长期预后相当。对于年龄因素，由于本研究队列患者偏年轻，可能会影响其对预后影响的分析。

在我中心已发表的HAD队列研究中，含中等剂量Ara-C的HAD方案相较标准剂量HAD方案可获得更高的1个疗程缓解率（87％对77％，*P*＝0.004）、3年无病生存率（58％对42％，*P*<0.001）和OS率（68％对59％，*P*＝0.014）。中剂量HAD方案是AML更强效的诱导化疗方案[15]。

综上所述，IDA联合标准剂量Ara-C与HAD方案均是AML患者有效的诱导化疗方案。本研究存在队列总样本量较小且为回顾性研究等不足，我们将在今后通过扩大样本量进一步完善分析。
